# Assessment on major browse feed resources and determine their chemical composition in Korhaye zone, Somali Region, Ethiopia

**DOI:** 10.1016/j.heliyon.2024.e40178

**Published:** 2024-11-07

**Authors:** Wondimagegn Tadesse, Kibebew Babege, Sale Wandara

**Affiliations:** Department of Animal Science, College of Dryland Agriculture, Kebri Dehar University, Ethiopia

**Keywords:** Browse, Chemical composition, Constraints, Feed

## Abstract

Assessment of major browse feed resources and determine the chemical composition of selected browse feed resource in *Korahay Zone, Somali Regional State, was the aim of this study*. Kebeles and the household heads were selected purposively in study area. The samples of fresh leaves (800 g) of selected browse species were collected to determine chemical compositions. SPSS version 20 was used for data analysis. The feed related constraints were ranked and analyzed by the ranking index. The current result indicated that, total of 22 trees, 6 shrub, 4 bush and 6 herbaceous species used as feed for livestock in dry and/or wet season were identified in the study area. DM, OM, CP, NDF, ADF, ADL and Ash of Dobera glabra, Grewia bicolor, Boscia minimfolia, Prosopis juliflora, Acacia Senegal, Unidentified (Alankhayir), Unidentified (Geed chic) were determined in current laboratory analysis. DM, OM, CP, NDF, ADF, ADL and Ash of Dobera glabra was 92.8 %, 94.28 %, 14.4 %, 42.5 %, 26.9 %, 5.22 % and 5.73 % respectively. Finally, feed quality and quantity, drought, invasive species and ecological deterioration were identified as major constraints of feed resources in study area. Due to this, intervention and further study should be conducted to tackle existing feed and feed related issues.

## 1 Introduction

Browse plants are one of the most cost-effective sources of feed for ruminant livestock [1]. During the dry season, when herbaceous vegetation dries out and deteriorates in quality and quantity, most browsing species maintain their greenness and nutritional content. According to Ref. [1] browsers can consume various parts of woody plants like leaves, twigs, flowers, pods and fruits. Browse plant species represent a valuable supply of feed and greatly contribute the nutritional need of livestock [2]. Browse refers to leaves and twigs from shrubs and trees available to ruminants as feed and in a broader sense including also flowers and fruits or pods.

Browse fodders represents cheap source of crude protein (CP) and minerals supplement to ruminants in the tropical region and are thus used as protein supplements to ruminants fed to low quality roughages due to their high content of CP (109–229 g/kg DM) and minerals, and their lower contents of fiber than mature tropical grasses [[3], [4]]. According to Ref. [3] browses had high levels of macro minerals: calcium, phosphorus, magnesium and sulphur and low to moderate levels of micro minerals: copper, molybdenum, iron, manganese, zinc and cobalt.

According to Ref. [5]*,* the nutritional contents of available feed resource is different based on species, verities, age of the plants, parts of the plant, soil types, climatic condition and agronomic practices. The browse species have greater potential in contributing towards alleviating the current feed shortage in the natural rangeland of the country. Several studies have noted the importance of browse plants in the arid and semi-arid areas of the world [6]. Likewise, conducting scientific research on available major browse feed source and there nutrient composition has paramount important to improve the productivity of livestock in arid and semi-arid environments like korahe zone of Somali regional state. However, limited study has been conducted on the available browse plant species and their nutritive value in korahe zone specifically Kabridahar and Lasdankhare districts. Therefore, it is important to gather data on the available browse feed source and their quality for communities rearing livestock particularly browsers and also for policy makers, researchers, stakeholders and bureau of livestock. Hence, this study was carried out with the aim of assessing the major browse feed resources and feed related constraints and also, evaluate their chemical composition in Korahe zone, Somali Region, Ethiopia.

## 2 Research Methdology

### 2.1 Description of the study area

Somali Regional State is the second largest region in Ethiopia with a land cover of 350,000 Kilometer Square. It is bordered with Oromia and Afar to the west, Djibouti to the north, Somalia to the north, east and south and Kenya to the southwest. Korahey is one of eleven administrative zones of the Somali region. Based on the 2007 Census conducted by the Central Statistical Agency of Ethiopia (CSA), this Zone has a total population of 312,713, of whom 177,919 are men and 134,794 are women. The largest ethnic group reported in Korahey was the Somali (99.22 percent); all other ethnic groups made up 0.78 percent of the population. Somali Language is spoken as a first language by 99.45 percent; the remaining 0.55 percent spoke all other languages. And also, 98.92 percent of the populations were Muslim.

The inhabitants of Korahey zone are predominantly pastoralists. On November 2003, the CSA conducted national agricultural census, which include the livestock census. For the Somali Region, the CSA generated estimated figures for the livestock population (cattle, sheep, goats, camels, and equines) and their distribution by commissioning an aerial survey. This national survey indicated that Korahey zone has 115,498 total number of camel and 4.7 number of camel per square kilometer which makes Korahey zone the second richest zone in camel population of the Somali region following Warder Zone.

**Fig. 1:**
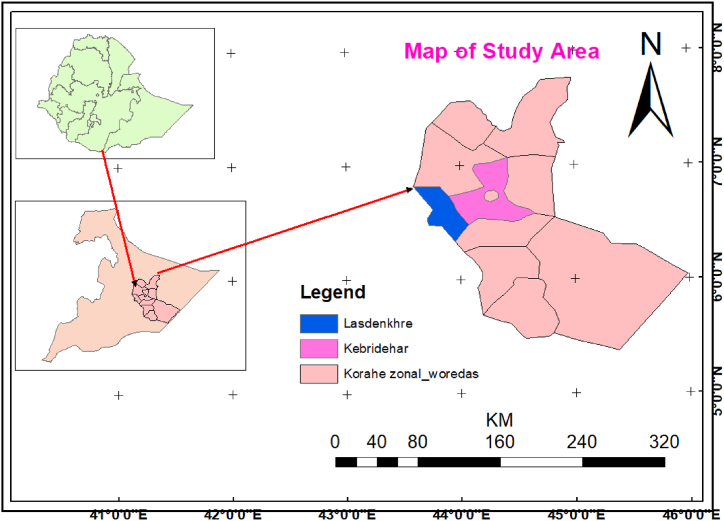
Map of the study area.

Korahey zone is located around 1030 KM from Addis Ababa, the capital city of Ethiopia. The topography of the study area is predominantly lowland plain with an average altitude of 493m above sea level with a few foothills of higher altitude. Korahey zone has a latitude and longitude of 6°44′25″N, 44° 16′38″E, respectively. The climate of Korahey zone is characterized as tropical semiarid in which temperature ranges from 23 to 36 °C. The area has bimodal rainfall pattern with two main rainy seasons in which the first is occur from mid-April to the end of June. The second rainy season occur from early October to late December. Kebri dehar and Lass-Dhankare are districts Located in the Korahe Zone of the Somali Region, Kebri dehar town has a latitude and longitude of 6°44′N 44°16′E and an elevation of 1609 m above sea level (see Fig. 1).

In the pastoral system, camel is becoming a leading animal because of the multipurpose role it has on the provision of milk, meat, social and cultural importance besides unpaid transport service to pastoral community. Camel is suitable in adapting to the changing climate and thriving under shortage of feeds and water and also means of utilizing rangeland feed resources that are not used by other species of livestock. Camel milk is an important component of human diet in many parts of the world.

### 2.2 Study design and procedures

The study was carried out in two parts namely survey and laboratory analysis. Survey part was conducted to assess available browse feed resources and feed related constraints in the study area while, in laboratory part, laboratory chemical analysis was conducted to determine the chemical composition of major browse feed resource which were collected after the survey. The major feed resources used for laboratory analysis were collected from previously surveyed kebeles of the study districts.

### 2.3 Data collection techniques and data sources for survey

The assessment was focused on the following major areas: general socio-economic characteristics of the households, the major browse feed resources, types of animal species and the major constraints related with livestock production in selected districts of korahey zone. Structured questionnaire and guided interviews were used to collect information from the respondents of the two selected districts in Korahey zone namely Kabridahar and Lasdankhare. In addition to this, observational studies were applied in the study areas to check validity of the data collected from structured questionnaire and guided interviews. To this, both Primary and secondary data from respondents’ and different offices were collected respectively.

The primary data sources were the respondents' (pastoralists’) during structured questionnaire and guided interviews. Moreover, interview with key informants and group discussion through respective selected focal persons in each district were the major data sources of this study. The secondary data was collected from zonal and district level agriculture and pastoralist offices. Sixteen [7] enumerators who knew the area and well acquainted with the culture and the local language will be selected and “trained” on the methods of data collection and the contents of the interview under close supervision of the researchers. The method of data collection employed was a single-visit formal survey [8].

### 2.4 Sampling technique and sample size determination for survey

The districts were selected purposively based on the availability or composition of major browse species of animal feed and other related criteria’. Purposive sampling procedure was implemented to select study kebeles because of difficulty to apply random sampling procedure due to the mobile, scattered and less accessible nature of pastoral communities and the household heads was selected based on livestock possessions, indigenous knowledge on name of browse species and willingness to be part of the survey. Out of the total kebeles located in the selected districts, 4 kebeles were purposively selected from each district (i.e. 4∗2 = 8 kebeles). Proportionally 30 respondents/pastoralists' were selected from each kebeles (i.e. 8∗30 = 240 respondents’) for this study.

### 2.5 Sample collection techniques and sample size determination for laboratory

Following the assessment, leaf parts of browse species sample were collected in the dry seasons (February–March 2022). All collected Leaf parts of collected browse species sample was labeled and dried for the analysis of chemical composition. Sample of same feed type was bulked to together on seasonal basis and then thoroughly mixed and sub-sampled following the method indicated by [9]. The leaf portion was sampled from major plants of selected browse species, weighted immediately after collection with digital sensitive balance and oven for dried at 65 for 72 h. For chemical analysis fresh leaves of each browse species weighing about 800 g was collected and then oven-dried. The oven dried samples were ground in a Willey Mill to pass through 1 mm sieve for the determination of chemical composition. Feed samples were analyzed for DM and ash using Official Methods of Analysis [10]. Nitrogen was determined using the micro Kjeldehl method. Crude Protein (CP) was calculated as N × 6.25. Neutral Detergent Fiber (NDF), Acid Detergent Fiber (ADF) and Acid Detergent Lignin (ADL) contents were analyzed by Van Soest method [11].

### 2.6 Data analysis

The data collected from each study sites were checked for any error and made correction before data analysis. Then, the collected data were coded and analyzed by using Software Package for Social Science version 26 (SPSS version 26). Moreover, the analyzed results were presented by using major descriptive statistics like; frequency, percentage, mean and standard error The constraints related to feed sources and livestock production was analyzed and presented by index. The indices is calculated as follows Sum of (3 for rank 1 + 2 for rank 2 + 1 for rank 3) given for an individual reason divided by the sum of (3 for rank 1 + 2 for rank 2 + 1 for rank 3).

## 3 Results

### 3.1 Major available browse species in the study area

The study has identified different available browse species utilized by camels and small ruminants in the study districts. The current study has identified twenty two [12] tree species, six [5] shrub species, four [3] bush species and six [5] herbaceous species (Table 1) as browse species for animals in dry, wet and both dry and wet season. Trees and shrubs are the most dominant woody species, becoming a major feed resource during dryness in the korahaye zone of Somali region by supplying protein and energy to maintain livestock production. The availability of plant species may vary from season to season and one district to another district. Trees and shrubs are a permanent source of feed for camels and small ruminants in dry season as compared to herbaceous species and herbaceous plants are less abundant in the dry season which decreases rapidly in quantity and quality after the rain.

**Table 1:** Available browse species in the study area

**Tree species**			
**Somali Name**		**Scientific Name**	
Mey-gaag	Boscia minimifolia	Mey-gaag	Boscia minimifolia
Miraqule	Prosopis juliflora	Miraqule	Prosopis juliflora
*Einjir*	*Euphorbia balsamifera Ait*	*Einjir*	*Euphorbia balsamifera Ait*
*Mal-mal*	*Commiphora molmol*	*Mal-mal*	*Commiphora molmol*
*Garas*	*Dobera glabra*	*Garas*	*Dobera glabra*
*Adaad*	*Acacia Senegal*	*Adaad*	*Acacia Senegal*
*Gob*	*Ziziphus mauritiana*	*Gob*	*Ziziphus mauritiana*
*Bur-bur*	*Carphalea glaucescens*	*Bur-bur*	*Carphalea glaucescens*
Chiic	Not identified	Chiic	Not identified
Alankhayir	Not identified	Alankhayir	Not identified
*Dhuya*	*Balbergia commiphoroides*	*Dhuya*	*Balbergia commiphoroides*
*Hagar*	*Commiphora agar*	*Hagar*	*Commiphora agar*
*Hareeri*	*Terminalla polycarpa*	*Hareeri*	*Terminalla polycarpa*
*Garbi*	*Acacia albida Del*	*Garbi*	*Acacia albida Del*
*Jeerin*	*Acacia edgeworthii*	*Jeerin*	*Acacia edgeworthii*
*Midha-fur*	*Boswellia neglecta*	*Midha-fur*	*Boswellia neglecta*
*Maanyo*	*Sonneratia alba*	*Maanyo*	*Sonneratia alba*
*Maraa*	*Acacia nilotica*	*Maraa*	*Acacia nilotica*
*Qansax*	*Acacia reficiens*	*Qansax*	*Acacia reficiens*
*Qudhac*	*Acacia tortilis*	*Qudhac*	*Acacia tortilis*
*Bur-bur*	*Carphalea glaucescens*	*Bur-bur*	*Carphalea glaucescens*
*Bil-il*	*Acacia mellifera*	*Bil-il*	*Acacia mellifera*
**Shrub Species**			
Dhebi	Grewia bicolour	Dhebi	Grewia bicolour
*Gomosh*	*Grewia villosa*	*Gomosh*	*Grewia villosa*
*Higlo*	*Cadaba heterotricha*	*Higlo*	*Cadaba heterotricha*
*Qalan-qal*	*Boscia coriacea*	*Qalan-qal*	*Boscia coriacea*
*Tiire*	*Clerodendrum Sp.*	*Tiire*	*Clerodendrum Sp.*
*Dhanfaruur*	*Grewia tenax*	*Dhanfaruur*	*Grewia tenax*
**Bush Species**			
*Gamo-dheere*	*Entada leptostachya*	*Gamo-dheere*	*Entada leptostachya*
*Kariiri*	*Solanum somalensis*	*Kariiri*	*Solanum somalensis*
*Adda-adeey*	*Sida ovate*	*Adda-adeey*	*Sida ovate*
*Balan-baal*	*Abutilon anglosomaliae*	*Balan-baal*	*Abutilon anglosomaliae*
**Herbaceous Species**			
Sarin	Cadaba ruspolii	Sarin	Cadaba ruspolii
*Wancad*	*Abutilon fruticosum*	*Wancad*	*Abutilon fruticosum*
*Madooya*	*Cadaba longifolia*	*Madooya*	*Cadaba longifolia*
*Saar*	*Coccinia grandis*	*Saar*	*Coccinia grandis*
*Baar*	*Hyphaene benadirensis*	*Baar*	*Hyphaene benadirensis*
*Maadathe*	*Dicoma somalensis*	*Maadathe*	*Dicoma somalensis*

### 3.2 Chemical composition of drought resistant browse species in the study area

The chemical composition of the major browse species available at dry season in the study area is presented in Table 2. The chemical composition of current study was focused on dry season available, drought tolerant and more selective for camel and small ruminant browse species. The CP content of the selected browse species were ranged from 10.08 to 16.20 % and the highest CP content (16.20 %) were recorded from *Acacia senegal*. The fiber of NDF and ADF content current analyzed browse species were ranged from 42.50 to 62.80 % and 26.90–44.20 %, respectively (Table 2).

**Table 2:** Chemical composition of major browse species in the study area.

		Chemical Composition						
**Somali Name**	**Scientific Name**	DM	OM	CP	NDF	ADF	ADL	Ash
*Dhebi*	*Grewia bicolor*	91.84	91.67	14.80	43.20	28.50	6.47	8.33
*Mey-gaag*	*Boscia minimfolia*	91.28	91.82	12.20	50.95	31.94	7.16	8.18
*Miraqule*	*Prosopis juliflora*	90.27	89.20	10.08	51.00	38.00	6.91	10.8
*Adaad*	*Acacia Senegal*	92.44	91.61	16.20	62.80	44.20	11.14	8.39
*Alankhayir*	*Unidentified*	90.58	91.05	14.10	56.19	38.82	10.25	8.95
*Garas*	*Dobera glabra*	92.80	94.28	14.40	42.50	26.90	5.22	5.73
*Geed chic*	*Unidentified*	91.28	89.93	13.60	59.18	41.52	10.84	10.07

### 3.3 Major constraints related to feed resource in the study area

Feed quality and quantity, drought, invasive species and ecological deterioration were some of identified feed related constraints in current study area (Table 3). Drought occurrence was one of the major feed related constraints in study area which recorded as first rank from existing problems. In pastoral area drought is a common problem due to unpredictable and eractic rainfall and ecological deterioration.

**Table 3:** Feed resource related constraints in study area.

Constraints	Woreda			
	Kebridahar		Lasdankhare	
	Index	Rank	Index	Rank
Feed quality and quantity	0.214815	4	0.222304	4
Drought	0.274074	1	0.287297	1
Invasive species	0.244444	3	0.230428	3
Ecological deterioration	0.266667	2	0.259970	2

Index = Σ of [3 × number of household ranked 1st+ 2 × number of household ranked2^nd^+ 1 × number of household ranked 3rd] given for particular valued feed source divided by Σ of [3 × number of household ranked 1st+ 2 × number of household ranked 2nd + 1 × number of household ranked 3rd + ….] summed for all valued feed source.

## 4 Discussion

### 4.1 Major available browse species in study area

The availability of different palatable plant species in the wet season diversifies alternative feeds for animals to browse. Although small ruminants and camels lack alternative feeds in the dry season and are forced to browse low-palatable feeds, feeds get a low chance to browse in the wet season. The decline of grazing pasture resources during the dry season forces small ruminants to consume more browse plants than other types of feed resources [13]. Feeding on browse species by small ruminants did not occur only during the dry season; rather, it was observed even at the peak of the wet season in the Jigjiga zone [13]. [14] reported that 21 indigenous browse species were identified from the three agro-ecologies of the Guba Lafto district, of which 11 were found in lowland, 9 in mid-altitude, and 7 were identified in highland areas. Most of the identified browse species in lowland and mid-highland were shrubs, while more tree species were observed in the highland. Based on the responses of participants, *Dobera glabra* tree species was the best and most dominant dry season feed resource for browsers at both Tree browse species like *Acacia tortilis, Acacia seyal, Acacia etbaica, Acacia nilotica, Acacia mellifera, Acacia brevispica, Acacia bussei, Balanites aegyptiaca, and Commiphora* species play a very important role as sources of feed primarily for browsing species such as camels and small ruminants [15].

The most widely utilized shrub species in the wet season were *Grewia villosa, Clerodendrum* sp.*, Boscia coriacea, and Grewia villosa. Grewia bicolor and Cadaba heterotricha were important shrub species widely utilized during the wet season***.** Camels and goats are more selective to browse shrubs than grass and herbaceous species in the wet season when feed is available. Some shrubs, like *Clerodendrum* sp. *and Boscia coriacea,* have medicinal value for animals and humans in addition to being a source of animal feed [16]. identified 12 locally important indigenous shrub species for camels in the Somali region of Ethiopia.

Bushes are less palatable and preferable camel feed sources compared with trees and shrubs in the wet season, although most of them are widely used as sources of feed in the dry season. It is considered a dry-season feed for small ruminants and camels since most of the bushy species have been perennials with evergreen phenology in the study area. Bushes are believed to be a dry-season safeguard for camels during prolonged dry seasons when there is climate change [16]. [7] also supported the idea that bush species were highly utilized by camels during the dry season when quality feed was absent. Some bush species, such as *Entada leptostachya and Abutilon anglosomaliae,* have medicinal importance for animals and humans in addition to being animal feed sources.

Herbaceous plants are preferred animal feed as compared with tree and shrub species during the wet season, and these plant species also play a great role in diversifying wet season camel and small ruminant feed. The most widely utilizable herbaceous plants were *Abutilon fruticosum, Coccinia grandis, Cadaba ruspolii, Cadaba longifolia, Dicoma somalensis*, and *Hyphaene benadirensis*. However, during the dry season, the herbaceous components are less abundant and often become more fibrous. As a result, livestock browse less palatable species [17].

The current result is consistent with the results of [18], who reported 47 indigenous browse species for Gamo Gofa and Wolayta, and [19], who reported 62 useful plants for Awash National Park [17]. also reported 18 different browse species identified as being important for different livestock classes in central rift valley areas of Ethiopia. Browse trees like Acacia nilotica, Acacia senegal, Acacia tortilis, and Acacia nubica are major browse species used as feed for small ruminants [20]. Similarly, Ziziphus spina-christi and Acacia species are not significantly preferred by cattle, especially when given a choice, according to Ref. [21].

### 4.2 Chemical composition of drought resistant browse species in the study area

According to Refs. [[5], [22]], the variation of the chemical composition of feed depends on the plant species, plant variety, soil type, plant fraction, plant management, and other factors. Also, variation in nutrient content among browse species may be due to soil fertility differences and the inherent ability of the plant to accumulate nutrients from the soil [12]. The results of the sampled species in laboratory analysis showed that there is variation in chemical composition from one species to another. The dry matter (DM) value ranged from 90.27 % to 92.8 % for the analyzed browse species. The highest value of DM (92.8 %) was recorded for Dobera glabra, while the lowest value (90.27 %) was recorded for Prosopis juliflora. The study entitled ′′The Potential of Camel Milk and Extracts of Major Plants Brown by the Animal for Diabetes Treatment showed that the DM content of Acacia Senegal was 90.02 %, which is slightly lower than the result of this study [23].

The DM content in the current study had lower ranges than those reported by (24) in camel-preferred browse species in Kenya, including Acacia Senegal. The study conducted in Babile Woreda of the Oromia region and Shinile of the Somali region indicated that Grewia bicolor contains 92.03 %, which is slightly comparable to the current study [23]. Besides this, a study conducted in the eastern Shawa zone of Oromia indicated that grewia bicolor contains 90.0 ± 0.05 DM, which is nearly in agreement with the current study [17]. The study conducted in the Afar region indicated that Dobera glabra had a 21.17 % ash content in the leaf, which is more than threefold that of the present study [24]. A study conducted by (25) indicated that Dobera glabra contained 90.88 % DM, which is slightly lower than the current study. The study entitled Identification and Nutritive Value of Potential Fodder Trees and Shrubs in the Mid-Rift Valley of Ethiopia indicated that grewia bicolor contained 9.2 ± 0.06 as h content [17].

The CP content of *Grewia bicolor* in the current study was 14.8%DM; it is slightly lower than the study conducted by (23). The CP content of *Dobera glabra,* regardless of age and composition, is 14.4%DM. The current laboratory analysis of Dobera *glabra* is in close agreement with the study conducted by (34). The CP content analysis of *Boscia minimfolia* is 12.2%DM. The CP content of *Acacia Senegal* is 16.6 % DM. The higher CP content of *Acacia Senegal has been* found in the current study when compared with the result obtained from a study conducted by (23). Other studies titled Chemical Composition and Digestibility of Preferred Forage Species by Lactating Somali Camels in Kenya indicate that the CP content of Acacia Senegal is slightly higher than the current study [25]. But the lower CP content of Acacia Senegal found in this study was lower than the study conducted in Shinell by (23). The CP content of two unidentified (*Geed chic* and *Alankhayir*) tree species was 13.6 % and 14.1 %, respectively. Overall, the current study's sample of browse species had CP contents that ranged from 10.08 to 16.20 percent, which was higher than the minimum of 7.25 percent needed to meet the ruminal microorganisms of sheep and goats and provide the minimum ammonia levels needed by rumen microorganisms to support optimal rumen activity [26].

The chosen browse species' NDF and ADF contents ranged from 42.50 to 62.80 % and 26.90–44.20 %, respectively. The soil type, plant species, age of the plant gathered for laboratory examination, and the portions of the plant harvested could all be contributing factors to the difference in fiber content of browsing species. Fiber fractions like NDF and ADF are important determinant factors of forage quality and digestibility [27]. The research studied by (17) showed that grewiabi contains a 54.7 ± 0.01 NDF value, which is higher than the present study. The NDF value greater than the threshold level 60 %, resulted in decreased voluntary feed intake, increased rumination time, and decreased conversion efficiency of ME [28]. Roughage diets with NDF content of 45–65 % and below 45 % were generally considered medium- and high-quality feeds, respectively [29]. According to (17), grewia bicolor has 41.9 ± 0.04 ADF values that are much greater than the current analyzed result. Roughages with less than 40 % ADF content are categorized as high quality, and those with greater than 40 % are categorized as poor quality [30]. Therefore, the range (26.90 %–44.20 %) of ADF and (42.50 %–62.80 %) of NDF content obtained from selected browse species in the current study were between the critical value of 45–65 % of NDF and below 45 % of ADF, which is generally considered a medium-quality forage for browsers.

### 4.3 Major constraints related to feed resource in the study area

One of the most unfortunate characters of Ethiopia's climate is great variability and erratic rainfall from year to year [31]. Ecological deterioration is next challenging problem for pastoralists because the ecosystem is fragile and improper management of feed resources have done sever problem for pastoralist. Gradual encroachment of range land with invasive species has created ecological deterioration in study area. Natural grazing land is deteriorating rapidly due to lack of attention especially in pastoral areas of the country [32]. Improper range land management creates both ecological crises and unconditional situation for pastoralists and livestock's.

## 5 Conclusion

In conclusion, the result suggested that the major browse species assessed in the study area during different seasons were twenty-two trees, six shrubs, four bushes, and six herbaceous plants, which are used as feed for livestock. Following the assessment, Grewia *bicolor, Boscia minimfolia, Prosopis juliflora, Acacia Senegal, Unidentified (Alankhayir), and Dobera glabra, Unidentified (Geed Chick),* were highly drought resistant and available during drought period and selected as major browse species in the study districts for the determination of DM, OM, CP, NDF, ADF, ADL, and Ash. Moreover, feed quality and quantity, drought, invasive species, and ecological deterioration were among the identified major constraints mainly related to feed resources in the study area. Therefore, further study should be done for more investigation of all available browse species based on season and to determine the chemical composition and anti-nutritional factors in the study area.

## CRediT authorship contribution statement

**Wondimagegn Tadesse:** Writing – review & editing, Writing – original draft, Visualization, Validation, Supervision, Software, Resources, Project administration, Methodology, Investigation, Funding acquisition, Formal analysis, Data curation, Conceptualization. **Kibebew Babege:** Writing – review & editing, Writing – original draft, Visualization, Validation, Supervision, Software, Resources, Project administration, Methodology, Investigation, Funding acquisition, Formal analysis, Data curation, Conceptualization. **Sale Wandara:** Writing – review & editing, Writing – original draft, Visualization, Validation, Supervision, Software, Resources, Project administration, Methodology, Investigation, Funding acquisition, Formal analysis, Data curation, Conceptualization.

## Disclosure statement

No potential conflict of interest was reported by the authors.

## Declaration of competing interest

The authors declare the following financial interests/personal relationships which may be considered as potential competing interests:Wondimagegn Tadesse Alem reports financial support was provided by Kebridehar University. Reports a relationship with that includes:. Has patent pending to. If there are other authors, they declare that they have no known competing financial interests or personal relationships that could have appeared to influence the work reported in this paper.
